# Cost analysis of two anaesthetic machines: "Primus^®^" and "Zeus^®^"

**DOI:** 10.1186/1756-0500-5-3

**Published:** 2012-01-04

**Authors:** Jose Hinz, Nadine Rieske, Bernd Schwien, Aron F Popov, Prashant N Mohite, Oliver Radke, Armin Bartsch, Michael Quintel, Klaus Züchner

**Affiliations:** 1 Department of Anaesthesiology, Emergency and Intensive Care Medicine, University of Göttingen, Göttingen, Germany; 2 Fachhochschule Nordhausen, Nordhausen, Germany; 3 Department of Thoracic and Cardiovascular Surgery, University of Göttingen, Göttingen, Germany; 4 Department of Cardiothoracic Transplantation & Mechanical Support, Royal Brompton & Harefield Hospital, Harefield, London, UK; 5 Department of Anaesthesiology and Intensive Care Therapy, University Hospital Dresden, Dresden, Germany; 6 Department of Critical Care & Anaesthesia, Royal Brompton & Harefield Hospital, Harefield, London, UK; 7 Robert-Koch-Str. 40, 37075 Göttingen, Germany

## Abstract

**Background:**

Two anaesthetic machines, the "Primus^®^" and the "Zeus^®^" (Draeger AG, Lübeck, Germany), were subjected to a cost analysis by evaluating the various expenses that go into using each machine.

**Methods:**

These expenses included the acquisition, maintenance, training and device-specific accessory costs. In addition, oxygen, medical air and volatile anaesthetic consumption were determined for each machine.

**Results:**

Anaesthesia duration was 278 ± 140 and 208 ± 112 minutes in the Primus^® ^and the Zeus^®^, respectively. The purchase cost was €3.28 and €4.58 per hour of operation in the Primus^® ^and the Zeus^®^, respectively. The maintenance cost was €0.90 and €1.20 per hour of operation in the Primus^® ^and the Zeus^®^, respectively. We found that the O_2 _cost was €0.015 ± 0.013 and €0.056 ± 0.121 per hour of operation in the Primus^® ^and the Zeus^®^, respectively. The medical air cost was €0.005 ± 0.003 and €0.016 ± 0.027 per hour of operation in the Primus^® ^and the Zeus^®^, respectively. The volatile anaesthetic cost was €2.40 ± 2.40 and €4.80 ± 4.80 per hour of operation in the Primus^® ^and the Zeus^®^, respectively.

**Conclusion:**

This study showed that the "Zeus^®^" generates a higher cost per hour of operation compared to the "Primus®".

## Background

Statutory funding changes in health care systems place hospitals under increasing cost pressures. Hospitals are encouraged to work more efficiently, reduce costs and adjust treatment protocols to secure their economic survival, which is essential in every healthcare system. The use of investment and cost-intensive medical and technical equipment in hospitals is discussed in terms of cost and profitability below. As the cost reduction in hospitals is a major objective, we conducted a cost analysis of two anaesthetic machines, the "Primus^®^" and the "Zeus^®^". Both of these machines are manufactured by Draeger. The "Primus^®^" anaesthetic machine allows for low-flow anaesthesia with fresh gas flow rates < 1 L/min. In addition to a manually controlled fresh-gas control, the "Zeus" has automatic oxygen control, medical air and volatile anaesthetics in a closed breathing system. The reason for having automatic control is to minimise the consumption of these gases, thus reducing the cost of anaesthesia during a surgical procedure. The aim of this study was to compare the "automatic" approach of the Zeus^® ^to the more typical operator-controlled method of the Primus^® ^and to investigate the financial implications of using these different anaesthetic machines in a clinical setting.

## Methods

The ethics committee of the University Hospital Goettingen (UMG) approved this study. We analysed the cost of two anaesthetic machines ("Primus^®^" vs. "Zeus^®^", Draeger AG, Lübeck, Germany). These anaesthetic machines use different types of fresh-gas control and were evaluated during surgery under general anaesthesia at the University of Goettingen. General anaesthesia (GA) in the "Zeus^®^" group was conducted with the "Zeus" (Draeger AG, Lübeck, Germany) anaesthetic machine. The "Primus^®^" group used the "Primus^®^" (Draeger AG, Lübeck, Germany) anaesthetic machine. The "Primus^®^" anaesthetic machine allows for low-flow anaesthesia with fresh gas flow rates < 1 L/min. In addition to the manually controlled fresh-gas control, the "Zeus^®^" has automatic oxygen control, medical air and volatile anaesthetics in a closed breathing system.

A total of 30 patients were included in this study and all were American Society of Anaesthetists (ASA) classification I-III. All the patients were also undergoing major cardiac or general surgery. Thirty patients were evenly randomised into either the "Zeus^®^" or "Primus^®^" group. Before the start of the study, the anaesthetic ventilators and circuits were checked with the manufacturer's recommended predetermined self-test. The maximum allowed measured leakage was limited to 25 ml. Oxygen and medical air consumption was calculated based on anaesthetic time. Connection of the patient to the anaesthetic machine was defined as the time period between the start time and the removal of the patient from the machine, which was defined as the end time. The induction phase was excluded from the study because it was performed with other anaesthetic machines in the anaesthetic room. The prices for the gas and vapour supplies of the studied devices were taken from our in-house pharmacies and equipment cost lists, and the prices for device-specific parts were taken from the Draeger price list. To calculate the hourly values for the anaesthetic machines, an annual workload of 1,920 h was calculated from equipment use of 8 h per day, 20 days per month.

### Anaesthesia

The specific anaesthesia protocol for each patient was determined by the anaesthetist overseeing the case. However, established clinical standards were always followed. After the patients were premedicated with 0.1 mg/kg of Midazolam (Dormicum ^®^, Roche, Basel, Switzerland) in the morning and evening, the induction of anaesthesia was performed intravenously with 2 μg/kg of Sufentanil (Janssen-Cilag, Neuss, Germany) and, if necessary, 0.3 mg/kg of Etomidate (Janssen-Cilag, Neuss, Germany) until the eyelash reflex was lost. Then, the patients were given 0.1 mg/kg of Pancuronium (Curamed Schwabe, Karlsruhe, Germany), followed by orotracheal intubation. Balanced anaesthesia was maintained with 1% Sevoflurane (Abbott, Wiesbaden, Germany) or 0.8% Isoflurane (Abbott, Wiesbaden, Germany), supplemented with a continuous infusion of an opioid (1 μg/kg/min of Sufentanil) during cardiac surgery and intermittent administration of an opioid (0.2 μg/kg of sufentanil) during general surgery [1]. In the general surgery cases, general anaesthesia was combined with a thoracic epidural. The mechanical ventilation was adjusted to achieve an end-tidal carbon dioxide (CO_2_) concentration of 35 mmHg and an oxygen saturation of 95%. This was achieved by adjusting the fraction of inspired oxygen and the positive end-expiratory pressure (PEEP). In the "Zeus^®^" group, the ventilator was used in the closed mode (auto control). In the "Primus^®^" group, the anaesthetist in charge was free to choose his or her dosage mode (high flow, low flow or minimal flow). Our clinical standards recommend that after an initial wash in phase with 4 l/min of fresh gas flow, a low-flow anaesthesia, with a fresh gas flow rate < 1 l/min, is used after reaching the desired steady state concentration of the volatile anaesthetic [2-4].

### Statistics

The statistical calculations were performed with the Statistica software (Statsoft Inc., Tulsa, OK, USA). For all the analytical methods, a p < 0.05 for alpha errors was considered significant. To determine if the results had a normal distribution, a Kolmogorov-Smirnov test was performed. Upon confirming a normal distribution, the results were expressed as the mean ± standard deviation. A paired Student's *t*-test was used to evaluate significant differences between paired samples, and an unpaired Student's *t*-test was used to evaluate differences between independent samples. In cases where the data were not normally distributed according to the Kolmogorov-Smirnov test, the results were presented as a median and a range. The test used to evaluate significant differences in the paired samples when the data were not normally distributed was the Wilcoxon matched-pairs test. For the independent samples that were not normally distributed, the Mann-Whitney *U *test was used.

## Results

The patient population did not differ in age, height, weight or operating time. No differences were observed with respect to volatile anaesthetics or the use of a thoracic epidural. In the "Primus^®^" group 13 patients received sevoflurane, and 2 patients received isoflurane. In the "Zeus^®^" group, 11 patients received sevoflurane, and 4 patients received isoflurane. A thoracic epidural was only used in the "Primus^®^" group (n = 2). All the cost comparison results related to anaesthesia for the "Primus^®^" and the "Zeus^®^" are summarised in Tables 1, 2, 3.

**Table 1:** Summary of maintenance costs for the "Primus^®^" and the "Zeus^®^"

Cost	**Primus**^® ^**(€)**	**Zeus**^® ^**(€)**
Maintenance 1 time/year (Draeger)	1000.90	1379.00

Maintenance 1 time/year (in-house)	420	420

	(120 + 300 spare parts)	(120 + 300 spare parts)

Vaporiser Maintenance	188	523.28

1 time/year (Draeger)	(94 × 2 Vaporisers)	(130.82 × 2 DIVA modules and 2 maintenance checks/year)

Vaporiser Maintenance	144	NA

1 time/year (in-house)	(72 × 2 Vaporisers)	

Total cost	1752.90	2322.28

**Table 2:** Cost comparison of two anaesthetic machines: "Primus^®^" and "Zeus^®^"

	Primus^®^	Zeus^®^	p-value
Age [years]	60 ± 19.6	72 ± 9.5	n.s.

Weight [kg]	80 ± 29.9	80 ± 29.4	n.s

Height [cm]	164 ± 30.8	163 ± 26	n.s

Purchase Costs [€]	62.894.01	88.056.43	

Minutes of operation [hour]/year	1,920	1,920	

Cost [€/hr of operation]	3.28	4.58	

Maintenance Draeger [€/year]	1.000.90	1.379.00	

Maintenance in-house [€/year]	420	420	

Draeger Vaporiser Maintenance [€/year]	188.00	523.28	

Vaporiser - in house maintenance [€/year]	144.00		

Maintenance [€/hr)	0.90	1.20	

Anaesthesia duration [minutes]	278 ± 140	208 ± 112	0.14

O_2_-consumption [m^3^/anaesthesia]	0.1468 ± 0.0846	0.233 ± 0.180	

O_2_-costs [€/anaesthesia]	0.0540 ± 0.030	0.086 ± 0.066	

O_2_-costs [€/hr]	0.015 ± 0.013	0.056 ± 0.121	< 0.05

Medical air-Consumption [m^3^/anaesthesia]	0.0546 ± 0.0377	0.0925 ± 0.06892	

Medical air-cost [€/anaesthesia]	0.02020 ± 0.01347	0.03144 ± 0.02264	

Medical air-cost [€/hr]	0.005 ± 0.003	0.016 ± 0.027	< 0.05

Volatile anaesthetic [ml/anaesthesia]	18.26 ± 9.71	27.10 ± 29.15	

Volatile anaesthetic [€/anaesthesia]	10.09 ± 6.43	9.67 ± 6.56	

Volatile anaesthetic [€/hr]	2.40 ± 2.40	4.80 ± 4.80	< 0.05

CO_2_-Absorber [€/hr]	1.36	1.36	

IBF-Filter [€/hr]		0.25	

Electricity cost [€/hr]	0.024	0.048	

Rent for an OP theatre [€/hr]	12.00	12.00	

Surface disinfection [€/anaesthesia]	0.15	0.15	

Consumable costs [€/anaesthesia]	17.03	17.03	

Training on equipment [€/hr]	0.54	0.54	

Cost of an anaesthetist [€/hr]	42.00	42.00	

Cost of an ODP [€/hr]	27.00	27.00	

Cost of a medical technician [€/hr]	24.00	24.00	

**Table 3:** Cost comparison of two anaesthetic examples of similar duration with the "Primus^®^" and the "Zeus^®^"

Anaesthesia costs	**Primus**^® ^**(€)**	**Zeus**^® ^**(€)**
**Example anaesthetic**		

Duration of anaesthesia [min]	120	116

Consumption v. A. [ml]	15.69	51.11

Air consumption [m^3^]	0.10	0.27

O_2 _consumption [m^3^]	0.03	0.16

**Cost Elements**		

Depreciation	6.55	8.87

Maintenance costs	1.83	2.33

Absorber cartridge costs	2.71	2.62

Cost filter for Zeus^®^		0.48

Electricity	0.05	0.09

Cost of anaesthetist	84.00	81.20

Cost of an ODP	54.00	52.20

Consumable costs	17.03	17.03

Disinfection	0.15	0.15

Medical air	0.04	0.10

O_2 _	0.01	0.06

Sevoflurane anaesthesia costs	9.27	30.19

Costs of training	1.08	1.05

Costs of rent	24.48	23.66

**Total cost [€/Example anaesthetic]**	201.19	220.03

### Cost

The studied anaesthetic machines, the "Primus^®^" and the "Zeus^®^", depreciate on a straight-line basis over a 10 year period in the UMG. Straight-line depreciation means that the cost of the depreciable asset will be divided equally among the years of its useful life. European list prices were used to reference the acquisition costs of the studied anaesthetic machines. Furthermore, maintenance expenditures were considered, which are necessary to maintain the anaesthetic devices in an operational state.

For the "Primus^®^", the acquisition cost was €62,894.01. Taking into account the 10 years of depreciation, the annual utilisation rate per hour was €3.28 for the "Primus^®^". The acquisition cost of the "Zeus^®^" was €88,056.43. Taking into account the 10 year depreciation period, the annual utilisation rate for the "Zeus^®^" was €4.58 per hour.

### Costs for device-specific accessories

The machines examined in this study required CO_2 _absorber cartridges (Draegersorb Clic 800 Plus, Draeger, Luebeck, Germany). These absorber cartridges were changed, on average, twice per week at a cost of €25 each. This led to an increase in operational cost of €1.36 per hour for the 1,920 h of service per year. The "Zeus^®^" anaesthetic machine required an additional IBF filter used in conjunction with the absorber cartridge. The IBF filters were changed twice per week, according to recommendations from Draeger, at a cost of €4.59 each. The annual cost of the filters used was €477.36, yielding costs of €0.25/h for the "Zeus^®^".

### Maintenance costs

The Medical Devices Act (MPG), the Medical Devices Operator Ordinance and proprietary inspection and maintenance regulations specify at what interval and how anaesthesia equipment must be checked. A maintenance contract exists between UMG and Draeger with alternating tasks. Draeger and the in-house Medical Technical Service (MTS) alternate performing the maintenance and safety checks once a year. This contract covers the maintenance costs of the devices, which were set at fixed prices for the 10-year period. These fixed amounts include staff costs from Draeger and spare parts costs. The replacement of specific parts is included in an annual service package. Therefore, these parts are always replaced, regardless of whether or not they show signs of wear. In-house MTS maintenance costs include medical technician costs. Because in-house maintenance cannot predict which parts need to be replaced, an amount of €300 was included based on experience in recent years.

Table 1 shows the composition of the maintenance costs for the "Zeus^®^" and "Primus^®^" anaesthetic machines.

### "Primus^®^"

For the "Primus^®^", a maintenance cost of €1,752.90/year was determined, which included the Draeger costs (€1,000.90) in addition to in-house maintenance costs (€120 per year). The cost of in-house maintenance was calculated from the hourly wage of a medical technician (€24 per hour) and the service time for the "Primus^®^" (approximately 5 h per year). Furthermore, a calculated cost of €300 for spare parts was expected. In addition to servicing the anaesthetic machine, the anaesthetic vaporisers are serviced separately. Because the "Primus^®^" operates with two anaesthetic vaporisers, the maintenance costs were doubled. The annual fixed maintenance cost for the vaporisers from Draeger was €94. The cost of in-house maintenance of the anaesthesia vaporisers was €72 per vaporiser. This €72 includes the hourly wage of a medical technician (€24/h) and a 3-hour maintenance period. Thus, the Primus^® ^service cost was €0.90/h in total.

### "Zeus^®^"

The total maintenance costs for the "Zeus^®^" were €2,322.28 per year. This amount comprised fixed costs by Draeger (€1,379), the cost of in-house maintenance (€120) and the calculated value for spare parts (€300). The "Zeus^®^" uses a vaporiser system called DIVA modules, which are special anaesthetic injection systems maintained exclusively by Draeger. The cost of maintaining a DIVA module by Draeger was €130.82 each and €523.28 per year. In total, the service cost for the "Zeus^®^" was €1.20/h.

### Energy costs

Energy cost is the electricity used to operate the equipment. Because a power consumption counter cannot be connected to the anaesthetic machine during operation for patient safety reasons, the power consumption of the anaesthetic machine was measured using a test lung. To calculate the electricity cost, a running time of 1,920 h/year was used. The "Primus^®^"electricity consumption was 130 W. At a price (as of May 2008) of 18.38 cents/kWh, the energy cost for the "Primus^®^" was calculated as €45.87 per year and €0.024 per hour. The "Zeus^®^" anaesthetic machine had a power consumption of 260 W. The electricity costs were €91.75 per year and €0.048 per hour for the "Zeus^®^".

### Cost of oxygen, medical air and volatile anaesthetics

Oxygen was delivered as a liquid (Air Liquide, Germany) and made available through a gas pipeline system to the entire University Hospital. The gaseous oxygen (€89.726 for 260,000 cubic meters of oxygen) yielded a cost of €0.37/m^3^, plus a maintenance cost of €5,712 per year. Medical air was produced by an in-house compressor and was provided through a medical gas pipeline. We were unable to determine the hospital's consumption of medical air and therefore set the price as identical to that of oxygen. Respective oxygen and medical air consumption was determined from the anaesthetic machines. Pre- and post-operative weighing of the anaesthetic vaporisers, on a precision balance (Sartorius, Sarorius, Göttingen, Germany), determined the consumption of the volatile anaesthetics. Consumption was calculated by dividing the difference in weight by the physical density of the anaesthetic. The physical density for sevoflurane and isoflurane is 1.53 g/ml and 1.50 g/ml, respectively. For 250 mls of sevoflurane and isoflurane, the cost is €147.67 and €31.10, respectively. This makes the final cost €0.5907/ml for Sevoflurane and €0.1244/ml for Isoflurane.

### "Primus^®^"

For the "Primus^®^", the average oxygen consumption was 0.147 ± 0.085 m^3 ^per anaesthesia, costing €0.054 ± 0.030. The average cost per minute for oxygen consumption was €0.015 ± 0.013/h. The average consumption of medical air per anaesthesia was 0.055 ± 0.036 m^3^, costing €0.020 ± 0.013. The cost per minute for medical air was €0.005 ± 0.003 per hour. The average consumption of volatile anaesthetics with the "Primus^®^" was 18.26 ± 9.71 ml per anaesthetic episode. This means that the average costs were €10.09 ± 6.43 per anaesthetic episode and €2.40 ± 2.40/h.

### "Zeus^®^"

The average oxygen consumption during anaesthesia with the "Zeus^®^" ranged between 0.233 ± 0.180 m^3^, costing €0.086 ± 0.066. Oxygen consumption for the "Zeus" costs €0.056 ± 0.121/h. The average air consumption with the "Zeus^®^" was 0.092 ± 0.069 m^3 ^per anaesthetic episode, and the average costs were €0.031 ± 0.023 per anaesthetic episode. The average cost per minute of air consumption amounted to €0.016 ± 0.027/h.

### Costs for surface cleaning of the anaesthetic machine

The surface disinfection of the equipment costs €0.15 per anaesthetic episode. This was calculated for a pack of disinfectant wipes containing 200 towels, which costs €10, assuming 3 cloths were used per cleaning.

### Training costs for the studied anaesthetic machines

For patient safety, it is necessary that all operators are knowledgeable about operating medical devices. The German legislature (MPBetreibV) stipulates that only personnel trained in the professional handling of medical devices are allowed to treat patients. Consequently, doctors, ODPs (operation department personal, called "anaesthetic nurses" in Germany) and nurses undergo regular in-hospital education on the handling of medical devices. The training is conducted and documented according to German legislature guidelines (§ 7 MPBetreibV) in the medical products directory for medical devices. For the hospital training costs, a flat rate of €60/h per teacher was used. For this study, a briefing time of 2 h for equipment training was assumed. Thus, for the lecturers, a total of €120 was calculated. To facilitate this training, the expenditure on telephone calls, equipment and room rent and other follow-up procedures for the event was assumed to be €200. In addition, during the time of training, staff costs for the participating anaesthetists and ODPs were considered. At an in-house training, six doctors and four nurses participated on average. The expense ratio is €42/h for an anaesthetist and €27/h for an ODP. The personnel costs thus totalled €720. The total cost of the equipment training amounted to €1,040 in total and €54/h.

### Costs for consumables

The cost of supplies used in this study consisted of a breathing circuit (€3.71), an endotracheal tube (€7.65), an HME-filter (€2.40) and a gas sampling line (€3.23). The ventilation system and the gas sample line were changed every day and the tube and the HME filters were changed between every patient. The supplies listed were used equally with both anaesthetic machines. Thus, the supply costs in this study were €16.99 per anaesthetic episode.

### Staffing costs for the anaesthetist

The 2008 budget for anaesthetists amounted to €5,499,450. This included social and health security, holiday pay and on-call commitment costs for 59.5 full-time employed anaesthetists, who worked 42 h per week. The annual working time of any staff group was calculated as follows: The standard hourly working time multiplied by 52 weeks per year. An anaesthetist therefore works 2,184 h per year, at a cost of €42 per hour.

### Staff ODP

Staffing costs per hour for the ODPs and medical technicians were calculated, which were €27 per hour for the ODPs and €24 per minute for the medical technicians.

### Discussion and Conclusions

Given the pressure on hospitals to decrease their costs, this work aimed to develop a methodology for comparing two anaesthetic machines ("Primus^®^" and "Zeus^®^"). In addition, the running costs of different fresh gas delivery modes were evaluated using a cost analysis.

The "Primus^®^" is able to use low-flow anaesthesia while the "Zeus^®^" utilises a quasi-closed automatic breathing circuit. The cost analysis has shown that the "Zeus^®^", with its automatic mode, was more expensive with respect to fresh gas and volatile anaesthetic consumption compared to manual metering with the "Primus^®^". Additionally, with respect to the cost of acquisition and maintenance, the "Zeus^®^" proved more costly than the "Primus^®^".

In addition to cost, the contribution of volatile anaesthetics to the destruction of the stratospheric ozone layer is of increasing importance, even if their effect on global warming is currently considered very low [5].

The cost of implementing and sustaining an anaesthetic service with inhalational anaesthesia using an anaesthetic machine can be divided into personnel costs, acquisition costs for the anaesthetic machine, maintenance costs, operating expenses, medical gases and volatile anaesthetic costs. The overwhelming majority of the expenses are staff costs [6]. The costs of the volatile anaesthetics are dependent on four factors:

(1) The cost per millilitre of liquid volatile anaesthetic

(2) The gaseous volume of the liquid from each millilitre of volatile anaesthetic

(3) The clinical potency of the volatile anaesthetic defined by the MAC value

(4) The anaesthetic machine selected fresh gas flow

A high fresh gas flow rate ("non-rebreathing system") prevents the rebreathing of anaesthetic gases and makes the control of the agent's concentration the simplest. However, this produces the highest costs due to wasted gases [7]. The economic benefits of low-flow anaesthesia are controversial [8,9]. Studies evaluating the cost effectiveness of anaesthetic machines most often look only at the consumption of fresh gases and volatile anaesthetics [10]. Although the costs of medical gases are only a small fraction of the total operational costs, it is still important to be aware of these costs. Aside from the cost of medical gases, it is also important to appreciate the costs incurred when using the anaesthetic machine and the dispensing mode in addition to the acquisition and maintenance costs.

Comparing acquisition and maintenance costs proved the "Zeus^®^" to be more costly than the "Primus^®^". At first glance, the increased purchase and maintenance costs are not surprising. The Zeus^® ^is an anaesthetic machine with the latest generation of technology. However, it is surprising that the quasi-closed automatic dispensing mode of the "Zeus^®^" is responsible for higher anaesthetic gas costs compared to the low-flow anaesthesia of the "Primus^®^".

The "Primus^®^" and the "Zeus^®^" differ in their fresh gas flow rates. Both the "Primus^®^" and the "Zeus^®^" have manual oxygen control, medical air and volatile anaesthetics to provide all types of fresh gas dosages (minimal flow, low flow and high-flow). The Zeus^® ^also offers an additional automatic control (TCATM = Target Controlled Anaesthesia) of oxygen, medical air and volatile anaesthetics in a quasi-closed breathing system, with the objective of minimising the consumption of fresh gases and volatile anaesthetics. The cost analysis of the "Primus^®^" and "Zeus^®^" anaesthetic machines has shown that the "Zeus^®^", in an automatic metering mode, was more wasteful compared to the manual metering mode of the "Primus^®^". At first glance, this is surprising. It was expected that the "Zeus^®^" would use less fresh gas and volatile anaesthetic because of its quasi-closed system. Even in the low flow mode, the anaesthesia machine would deliver surplus fresh gas and anaesthetic into the breathing circuit. This effect is based on the fresh gas control system in the "Zeus^®^" anaesthetic machine.

Because of the automatic control of fresh gas and volatile anaesthetic flow, a change in the concentration of volatile anaesthetic in the circle system was achieved utilising maximum fresh gas flow rates of oxygen and/or compressed air. This means that during these rapid adjustments to the desired concentration of volatiles in the circle system, the breathing circuit is changed to a quasi open-circuit system, with high losses of fresh gas and volatile anaesthetic. Furthermore, it has become apparent that the "Zeus^®^", after working for some time at a constant inspiratory concentration of volatiles in a circle system, intermittently rinses the circuit system with high fresh gas flow rates.

This may prevent unexpected increases in nitrogen in the circle system. In a study of 44 patients undergoing minor urological surgery, the consumption of fresh gas and the volatile anaesthetic desflurane were higher in the group with the automatic closed mode of the "Zeus^®^", as compared to the mode with a special low-flow protocol [11]. Studies in vitro and in vivo showed that the consumption of carrier gases and volatiles can be reduced if the "Zeus^®^" is operated in an automatic dispensing mode and if the maximum fresh gas flow rates during the rinse phases were limited to 1-6 l/min [12,13].

Table 3 shows the cost of inhalational anaesthetics using different machines for the same duration of anaesthesia. In addition to the cost of the fresh gas and the volatile anaesthetic acquisition, maintenance, operational and personnel costs were also included in the calculation. This method of anaesthesia equipment cost analysis allows for the comparison of different anaesthesia devices and the discovery of potential cost savings. It also evaluates the percent of the anaesthetic gases used in the total cost, especially in the acquisition and maintenance costs, which are only a small proportion of the overall costs of a two-hour anaesthesia. The higher amortisation of the acquisition and maintenance costs savings in anaesthetic gases is not possible with the current hardware and software platforms. Contrary to our initial assumptions, the "Zeus^®^" had higher anaesthetic gas costs with the tested hardware and software.

### Limitations

This cost analysis was conducted in theatres where elective surgery was performed. The local hospital guidelines recommend the use of low flow anaesthesia for the "Primus^®^" and the automatic dosing mode when using the "Zeus^®^". In-house guidelines recommend the use of 1% Sevoflurane concentrations during cardiac cases when cardiopulmonary bypass is used. To our knowledge, during this study all the anaesthetists followed the house intern recommendations. However, it is likely that the unblinded anaesthetists in the Primus^® ^group unconsciously modified their usual behaviour because of their participation in this study to achieve reduced costs. Therefore, this study may suggest that the attentive anaesthetist can achieve economy equal to or better than an automated system.

As is customary when conducting a study such as this, we had to make assumptions for some cost categories because there is no empirical data for these cost categories. Because the same assumptions were made for both anaesthetic machines, a biased error would affect both anaesthetic machines equally. The gas consumption for oxygen and compressed air was taken from the logbook of both devices. Currently, we are investigating other anaesthetic machines at UMG. The "Zeus^®^" is receiving a software update to version 4, and the pneumatically operated anaesthetic machine "Leon plus" ("Heinen and Löwenstein") has been submitted to a cost analysis. Questions, such as whether powering anaesthetic machines with compressed air is more economical than powering with electricity, can be addressed in this manner.

In summary, we conclude that it is possible to standardise the cost analysis for anaesthetic machines using a standard method to compare acquisition, maintenance and running costs. With the current software, the "Zeus^®^", in the automated dosing mode, generates higher costs per working hour than the "Primus^®^" in the manual low flow mode per working minute. Further studies are needed to evaluate how the updated version 4 software effects the consumption of oxygen, medical air and volatile anaesthetics in the "Zeus^®^".

## Competing interests

This study was supported by departmental funding and the medical supply company Draeger (Draeger AG, Lübeck, Germany).

## Authors' contributions

JH conceived the study, was responsible for ethics submission, grant and wrote the manuscript. NR and BS participated in the study design, and data collection. AFP, PNM, OR and AB helped with drafting the manuscript. MQ and KZ added important comments to the paper. All authors read and approved the final manuscript.
